# Citrus pectin-coated inhalable PLGA nanoparticles for treatment of pulmonary fibrosis†

**DOI:** 10.1039/d4tb01682c

**Published:** 2025-01-27

**Authors:** Kalindu Perera, Moez Ghumman, Parand Sorkhdini, Carmelissa Norbrun, Seraphina Negash, Yang Zhou, Jyothi U. Menon

**Affiliations:** a Department of Biomedical and Pharmaceutical Sciences, College of Pharmacy, University of Rhode Island Kingston RI 02881 USA jmenon@uri.edu; b Department of Molecular Microbiology and Immunology, Brown University Providence Rhode Island 02912 USA; c Department of Cell and Molecular Biology, University of Rhode Island Kingston RI 02881 USA; d Department of Chemical Engineering, College of Engineering, University of Rhode Island Kingston RI 02881 USA

## Abstract

Pulmonary fibrosis (PF) is a chronic interstitial disorder of the respiratory system that can be debilitating as it progresses and has experienced a slow rise in incidence in past years. Treatment is complicated by the complex aetiology of the disease and the off-target effects of the two FDA-approved therapeutics available on the market: pirfenidone and nintedanib. In this work, we propose a multipurpose nanoparticle system consisting of poly(lactic-*co*-glycolic) acid polymer (PLGA) and a coating of citrus pectin (CP) for galectin-3 targeting and anti-fibrotic therapy. Pectin from citrus peels has been observed to have anti-fibrotic activity in a range of fibrotic tissues, causing a decrease in the expression and activity of galectin-3: a key, upregulated marker of fibrosis. We show that the CP-PLGA nanoparticles (NPs) have an average diameter of 340.5 ± 10.6 nm, compatible with inhalation and retention in the deep lung, and that CP constitutes, on average, 40.3% of the final CP-PLGA formulation. The NPs are well-tolerated by MRC-5 lung fibroblasts up to 2 mg mL^−1^. We demonstrate the NPs’ ability to target transforming growth factor β (TGFβ)-treated fibrotic MRC-5 cells in a specific, dose-dependent manner, saturating at approx. 250 μg mL^−1^*in vitro*, and that our NPs have potent anti-fibrotic activity *in vivo* in particular, reversing bleomycin-induced fibrosis in mouse lungs, accompanied by marked reduction in profibrotic markers including collagen 1, fibronectin, α-smooth muscle actin, β-catenin and galectin-3. In all, we present an inherently therapeutic inhalable nanocarrier for galectin-3 targeting and anti-fibrotic therapy. We envision this carrier to be doubly effective against fibrotic lung tissue when combined with an encapsulated anti-fibrotic drug, improving overall/total therapeutic efficacy and patient compliance *via* the reduction of off-target effects and additive therapeutic effects.

## Abstract 2

Citrus pectin coated PLGA nanoparticles can significantly ameliorate pulmonary fibrosis over 24–72 h *in vivo*.

## Graphical abstract

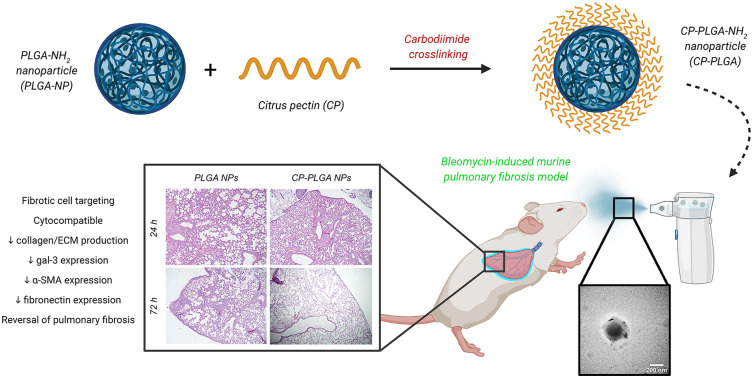

## Introduction

**Fig. 1:**
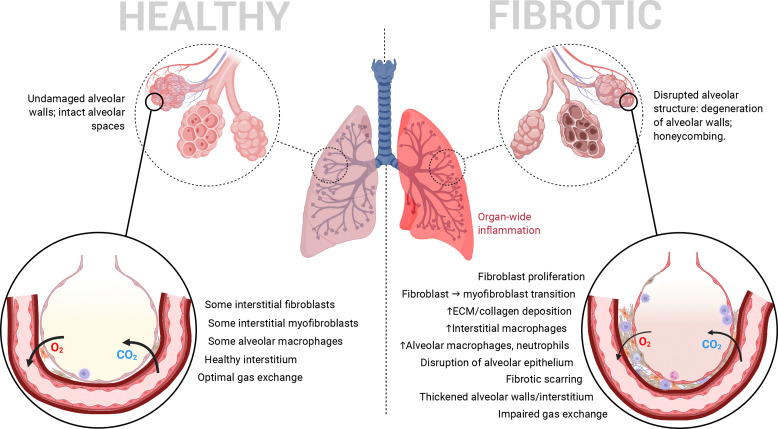
Pathological features of the deep lung when healthy *vs.* when afflicted by PF. Heavy fibrotic scarring and proliferation of myofibroblasts causes progressively deteriorating alveolar structure/organization and impairs pulmonary function by impeding efficient gas exchange.

Pulmonary fibrosis (PF) is a chronic form of interstitial lung disease (ILD) that distorts the lung architecture and impairs respiration.^1,2^ The incidence and prevalence of this condition, particularly its idiopathic form, are higher in the developed world, especially Western Europe, North America and East Asia, most commonly in adults aged 65–70.^3^ It is characterized by a steady decline in lung function, caused by progressive scarring (*i.e.*, development of fibrosis) in the lung parenchyma, particularly in alveolar/perialveolar tissue (causing alveolar septal thickening in particular). This hinders efficient gas exchange across the alveolar–capillary interface and leads to traction bronchiectasis and alveolar remodelling.^3,4^ High levels of inflammation in the lungs are another common hallmark of PF as in many other ILDs.^4,5^ In combination, these pathophysiological features (summarized in Fig. 1) result in progressively worsening lung function, commonly quantified by the decline in forced vital capacity.^4,6,7^ PF's influence can eventually cross organ systems *via* common complications such as pulmonary hypertension and respiratory failure.^7^ Interestingly, recent work surrounding SARS-CoV-2 has identified PF as a long-term complication of severe COVID-19 as well.^8,9^

Currently, pirfenidone (PFD) and nintedanib (NIN) remain the sole FDA-approved drugs indicated for use in patients with PF, although their adoption in clinical practice in the United States has decreased over time since their approval in 2014.^10^ This decreased adoption can primarily be attributed to the high cost of both. In addition, they are both administered orally several times a day in large doses (PFD: 801 mg day^−1^,^11^ NIN: 300 mg day^−1^ (ref. 12)), and cannot cure or reverse the disease, although they are useful for mitigating symptoms or slowing disease progression. A notable reason for the chronic, high-dose administration of both drugs is the high level of hepatic metabolism they undergo, necessitating high dosages for achieving reasonable levels of bioavailability.^13^ This increases the potential of side effects and the varying levels of hepatotoxicity associated with both drugs.^14^ Thus, chronic oral dosing of these antifibrotic agents can result in systemic side effects, greater out-of-pocket expenses, and poor patient adherence/compliance.^15^ Alternative approaches for targeted and localized treatment of PF are therefore needed. A more in-depth review of the pharmacological mechanisms of existing PF-targeted therapies, possible novel therapeutics and targets, and directions for future formulation approaches can be found in a recent review published by our group.^16^

Galectin-3 (gal-3) is a prominent β-galactoside-binding lectin secreted primarily by macrophages and fibroblasts that has been found to play a key role in a range of fibrotic diseases, including PF.^17–21^ It was previously shown to be upregulated in other fibrotic settings such as liver and renal fibrosis, with recent studies showing it to be overexpressed in PF as well.^22^ Levels of gal-3 are elevated in the bronchoalveolar lavage fluid (BALF) of PF patients relative to healthy individuals.^23^ Furthermore, elevated serum levels of galectins-1, 3, and 9 have been found in patients with PF and idiopathic nonspecific interstitial pneumonia (NSIP) relative to healthy controls.^23^ Gal-3 exerts profibrotic properties through interactions with transforming growth factor β1 (TGFβ1) signalling and by encouraging and enhancing cell–ECM adhesion.^18,22,24^ Moreover, in what may be a dangerous positive feedback loop, it is capable of stimulating fibroblast differentiation and collagen synthesis in both a TGFβ1-dependent and -independent manner.^18^ In all, this makes gal-3 a promising therapeutic target for fibrotic lung diseases. This potential approach was recently explored through the development of a gal-3 inhibitor, TD139/GB0139: a synthetic thiodigalactoside that was undergoing clinical trials as a potential treatment for PF.^25^ However these studies have since been halted due to the drug's inability to show target engagement in patients. Herein, we report the use of citrus pectin (CP), a naturally occurring galactoside analogue found within the peels of citrus fruits, as an alternative gal-3 inhibitor for pulmonary fibrosis treatment. It has been the subject of study in recent years for its anti-inflammatory as well as anti-fibrotic properties.^26–28^ CP's mode of action against gal-3 is twofold: firstly, it binds competitively to the lectin, impairing its ability to get involved in the fibrotic signalling process; secondly, through a process that is as-yet poorly understood, it has been shown to be capable of downregulating gal-3 production.^27,29^ Mouse studies with modified citrus pectin (a water-soluble form of CP) have also shown its potential to reduce macrophage infiltration into tissues (and, by extension, reduced levels of pro-inflammatory cytokines).^30,31^

As noted previously, the delivery of existing drugs targeting PF encounters problems with bioavailability and off-target effects. Given this, and the localized nature and relatively easily accessible location of PF in the body, the pulmonary route of administration becomes an attractive one.^32^ Indeed, trials are ongoing to examine the possibility of inhaled delivery of PFD and NIN; while these have shown some promise, the dosing regimen appears to remain more or less the same- either once or twice daily.^33–35^ In this context, another approach to increase the therapeutic efficacy and reduce the overall toxicity of antifibrotic agents is to encapsulate them within a nanocarrier. In general, nanoparticle drug delivery systems enable sustained or controlled drug release by acting as reservoirs of therapeutic agents and protecting encapsulated agents from degradation while also prolonging circulation/tissue retention time through reduced clearance.^36,37^ More specifically, inhalable nanoparticles are capable of deep lung penetration and provide localized therapy with less frequent dosing and non-invasive delivery to increase patient compliance.^37,38^ We have previously shown that poly(lactic-*co*-glycolic) acid (PLGA) nanoparticles containing different therapeutic agents, when nebulized into anesthetized, intubated rats, accumulated in the deep lung tissue, where they were retained for 7–10 days.^39,40^ The use of nano- (and micro-) scale carriers for inhaled delivery of pirfenidone is not new; several groups have previously explored this possibility and concluded that encapsulation within a nanoscale carrier greatly enhanced therapeutic efficacy and potential dosing frequencies compared to freely inhaled drug.^41–44^ Nanoparticle drug delivery systems also offer the opportunity to encapsulate multiple therapeutic agents within a single formulation for combination treatment through multi-layered or coated particles, or through modification of the particles’ surface or (in the case of materials such as dendrimers) the nanocarrier matrix itself.^45^

In this work, we present a formulation of CP-coated PLGA nanoparticles for the treatment of PF, seeking to ameliorate the fibrotic effects of the disease through CP/gal-3 mediated intervention. This delivery platform has the potential to act as a carrier for traditional anti-fibrotic drugs such as PFD and NIN in a far more directed/targeted and controlled manner.

## Experimental

### Materials, cells and animals

Unless otherwise noted, all chemicals were purchased from Thermo Fisher Scientific (Waltham MA, USA). Amine-terminated poly(lactide-*co*-glycolide) (PLGA-NH_2_·HCl; 10k MW, 50 : 50 lactic : glycolic acid) was purchased from Nanosoft Polymers (Winston-Salem NC, USA). Polyvinyl alcohol (PVA; MW 13 000–23 000, 87–89% hydrolyzed), 2-(*N*-morpholino) ethanesulfonic acid (MES), phosphate buffered saline (PBS) tablets, dimethyl sulfoxide (DMSO; ≥99.5%), methanol (>99.8%), Triton X-100, DAPI, paraformaldehyde, acrylamide, *N*,*N*′-methylenebisacrylamide and ammonium persulfate were purchased from Sigma-Aldrich (St. Louis MO, USA). Sodium dodecyl sulfate was purchased from Invitrogen. Nile Red dye was purchased from TCI (Portland OR, USA), while 1-ethyl-3-(3-dimethylaminopropyl)-carbodiimide (EDC) and citrus pectin (CP) were purchased from Alfa Aesar (Haverhill MA, USA). TD139 was purchased from Selleck Chemicals (Houston TX, USA), and recombinant human tissue growth factor β1 (TGFβ1) was purchased from R&D Systems (Minneapolis MN, USA) Antibodies for western blotting were obtained as follows: anti-galectin-3 (Invitrogen), anti-α-SMA and -vinculin (Abcam), and anti-β-catenin (SantaCruz). Polyvinylidene fluoride (PVDF) membrane, loading-/running gel buffers and Tween-20 were purchased from Bio-Rad (Hercules CA, USA), and an ECL Western blot analysis kit was purchased from Cytiva (Marlborough MA, USA). A549 human lung cancer epithelial cells and MRC-5 human lung fibroblasts were purchased from the American Type Culture Collection (ATCC; Manassas VA, USA). WT C57BL/6 mice were purchased from The Jackson Lab (Bar Harbor ME, USA).

### Nanoparticle formulation

Nanoparticle (NP) synthesis was carried out using a modified single emulsion method as described previously.^46^ In brief, an oil phase consisting of a 20 mg mL^−1^ solution of PLGA-NH_2_ in chloroform was dropped into a 10 mL surfactant solution of 5% (w/v) PVA in ultrapure water under constant stirring. The resultant crude emulsion was subjected to pulsed probe sonication using a FB120 Sonic Dismembrator (Fisher Scientific, USA) for 5 minutes. The emulsion was then left stirring overnight to allow for solvent evaporation, following which the NPs were collected and purified through two 30 minute ultracentrifugation steps (25 000 rpm) in an Optima L-100 XP ultracentrifuge (Beckman Coulter, USA). Particles were then frozen and subjected to lyophilization. For uptake studies, a fluorescent dye–Nile Red–was dissolved directly in the oil phase at 100 μg mL^−1^ prior to addition to the water phase.

### Citrus pectin conjugation

Carbodiimide crosslinking chemistry was employed for conjugation/coating of CP to the preformed PLGA NPs. A mass ratio of 1 : 10 : 10 : 1 NPs : EDC : NHS : CP was used, and the reaction carried out in MES buffer (0.5 mL per mg NPs, prepared in ultrapure water) at an approximate pH of 5.0. EDC and CP were combined in the appropriate volume of MES buffer and shaken at room temperature (RT) for 30 minutes. NHS was then added, and the reaction shaken for a further 30 minutes, following which the lyophilized NPs were added and the mixture was shaken overnight. NPs were then collected through ultracentrifugation and lyophilization as outlined previously.

### Physical characterization of nanoparticles

#### Particle size and zeta potential

The size (*via* dynamic light scattering, DLS) and surface charge of the nanoparticles were measured (*n* = 3) using a Zetasizer Nano ZS (Malvern Panalytical, UK). Samples for sizing/zeta potentiometry (ZP) were prepared by dispersing NPs in 1–1.5 mL of ultrapure water to a count rate of 200–300 kcps. Scanning electron microscopy (SEM) of particles pre- and post-CP coating was performed using the secondary electron detector of Sigma VP field emission SEM (Zeiss, Germany) at an electron accelerating voltage of 5 kV. Samples for SEM imaging were prepared by drying of a dilute drop of NP suspension in ultrapure water on a silicon wafer secured on a SEM stub using carbon tape, followed by sputter-coating with gold.

#### Confirmation of CP coating

Attenuated total reflectance Fourier-transform infrared (ATR FT-IR) spectroscopy and transmission electron microscopy (TEM) were first carried out to confirm CP coating of/conjugation to the NPs. IR transmittance spectra were acquired in the 400–4000 cm^−1^ range at a 2 cm^−1^ spectral resolution and 24 scans per sample, by means of an IRTracer-100 spectrometer (Shimadzu, Japan). For FT-IR, samples were desiccated prior to analysis to avoid interference by trace quantities of water. Brightfield TEM was carried out using a JEM-2100 TEM (JEOL, Japan); samples were prepared by placing and air-drying a drop of dilute NP suspension on a copper TEM grid. Further confirmation of conjugation was evaluated *via* thermogravimetric analysis (TGA) using a TA instruments TGA 55 (Waters, USA), over a temperature range of 25 to 500 °C at a ramp of 10 °C within a nitrogen atmosphere.

UV-vis absorbance spectrometry (*λ* = 325 nm; *n* = 3) was used to quantify the amount of CP conjugated to NPs, using the ferulic acid groups inherent to the CP's structure (see the ESI,† Fig. S1) to aid in this effort as has previously been described in the literature.^47^ Briefly, a calibration curve was drawn up with known concentrations of CP in a 50:50 mix of acetonitrile/ultrapure water, followed by absorbance measurements. CP-PLGA NPs were dispersed in the same solvent mix, allowed to shake for 6 hours and subjected to absorbance measurements. The concentration of CP in the NP sample was calculated by comparison with the calibration plot (see the ESI,† Fig. S2).

#### Stability in biorelevant media

CP-PLGA NPs were suspended in triplicate in either PBS (pH 7.4) or simulated lung fluid/Gamble's solution.^48^ These suspensions were allowed to shake at 37 °C over a period of 7 days, with DLS and ZP measurements being collected daily.

#### *In vitro* experiments

All cells were grown in Dulbecco's Modified Eagle Medium (DMEM) supplemented with 10% fetal bovine serum (FBS) and 1% penicillin/streptomycin and maintained in standard culture conditions (37 °C, 5.0% CO_2_) unless otherwise indicated. All cell detachment steps were performed using 0.5% trypsin-EDTA.

#### Cytocompatibility assays

All cytocompatibility studies were performed by means of the MTT cell viability assay. Cells were plated on 96-well plates at a density of 5000 cells per well, then treated with the appropriate concentration of treatment agent. Incubation was performed for 24 hours, following which cells were washed gently with PBS and MTT-containing media was placed in the wells. A 2 h incubation under standard culture conditions followed, after which the media was again aspirated, 100 μL per well of DMSO was added, and absorbance measured at 570 nm. The cytocompatibilities of CP-PLGA NPs (0–2 mg mL^−1^) and free CP (0–10 mg mL^−1^) were assessed on both A549 and MRC-5 cell lines. A comparative cytocompatibility study of CP *vs.* TD139 on MRC-5 cells (0–2 mg mL^−1^) was carried out as well. All treatments were performed at *n* = 4.

#### Nanoparticle uptake study

MRC-5 cells were plated at a density of 10 000 cells per well on 96-well plates. The cells were then starved using serum-free DMEM for 12 h, following which NP treatment groups (encapsulating dye, ±CP coating, 0–1 mg mL^−1^) were added to the wells in low-serum (1% FBS) DMEM (LSM) with or without the fibrotic agent TGFβ1 at a concentration of 10 ng mL^−1^ as described previously.^49–51^ The cells were incubated under standard culture conditions for 2 hours, after which the treatment media was aspirated, cells were gently washed twice with PBS, and 250 μL per well 1% Triton X-100 in PBS was added to all treated wells. The wells were gently scratched, and the plates were left overnight at 4 °C to allow for cell lysis. The plates were then warmed to RT, and the absorbance at 550 nm was measured, alongside a standard series of dye-encapsulating NPs. Aliquots of the lysate were employed for a BCA assay to measure per-well protein content, carried out following the manufacturer's instructions. Results were reported as micrograms of NP uptake per microgram of cellular protein. All treatment groups and standards were plated at *n* = 4. The above was repeated with A549s at the same cell density in order to model non-fibroblast uptake of the NPs.

Representative images of NP uptake by MRC-5s were obtained using EVOS microscopy. In brief, cells were treated as outlined previously, washed, fixed using 4% paraformaldehyde in PBS for 10 min, counterstained with DAPI and subjected to imaging.

#### Western blotting

MRC-5 cells were plated at 20 000 cells per well in 6-well plates, following which starvation was carried out as outlined in previously. Treatment groups prepared in TGFβ1-containing (5 ng mL^−1^) LSM were added to appropriate wells (healthy controls treated with plain LSM), and the cells were incubated under standard culture conditions for 24 h. Treatment media were then aspirated, and the wells were washed twice with PBS. Cold RIPA lysis buffer with a protease/phosphatase inhibitor cocktail was used to perform protein extraction, alongside probe sonication (30 s, 50% amplitude, 5 s pulses) on ice. Total cell protein was isolated from lysates following centrifugation at 4000*g* for 10 min at 4 °C and subjected to quantification *via* BCA assay.

Western blotting was employed to determine the efficacy of the treatment groups in ameliorating fibrosis by measuring the levels of three marker proteins, relative to untreated, healthy controls. Following denaturation at 90 °C for 3–5 minutes and cooling on ice for the same length of time, lysate samples were loaded onto 10% acrylamide gels and electrophoresed at 85 V for 30 min, then at 110 V for 1 h. Transfer to PVDF membranes was performed at a constant 0.3 A for 3 h. Transfer was confirmed by Ponceau S staining, and strips were cut to isolate protein bands of interest. Strips were subjected to blocking with 5% w/v fat free dry milk in TBS-T for 2 h, then probed with appropriate primary antibodies at manufacturer-recommended concentrations overnight at 4 °C on an orbital shaker. Strips were washed and re-probed with appropriate secondary antibodies for 2 h at 4 °C with shaking, following which they were washed and subjected to ECL staining. These were placed in development folders and imaged immediately using the chemiluminescent imaging option on an iBright 750 imager (Invitrogen, USA) at the optimal exposure time. Quantification of band intensity was performed using ImageJ, and results reported as loading control-normalized intensity.

#### RT-PCR

Cells were treated as outlined for western blotting, and lysis was performed following manufacturer instructions in TRIzol reagent. Total RNA was extracted using the QIAGEN RNeasy kit, and cDNA was synthesized from the RNA using the Bio-Rad iScript cDNA synthesis kit. All kits were used per the manufacturer's instructions. mRNA levels for collagen 1a-1, collagen 3a1, and fibronectin were measured using RT-PCR. Primer sequences for these genes were obtained from the PrimerBank of Massachusetts General Hospital and are provided in the ESI† (Table S1).

### *In vivo* evaluation of nanoparticle efficacy

WT C57BL/6 mice were utilized for all experiments described. These mice were treated with either PBS or bleomycin (15 U kg^−1^) in PBS by IP injection in a total volume of 100 μL. This treatment was repeated daily for six consecutive days to induce fibrosis. On day 12, mice were intratracheally treated with PBS, free CP (0.1 mg in 50 μL), or 0.25 mg of either PLGA-NH_2_ NPs (PLGA) or CP-PLGA NPs. Lungs were harvested 24 h and 72 h after nanoparticle treatments. The left lung was fixed with formalin for H&E histological evaluations. The superior lobe was lysed in TRIzol reagent and total cellular RNA was extracted by Qiagen RNeasy kit. cDNA was synthesized using the BioRad iScript cDNA synthesis kit and used for measurements of α-smooth muscle actin, collagen 1-a1, and fibronectin (for primer sequences, see the ESI,† Table S1). The middle lobe was snap frozen for protein extraction to measure gal-3 protein levels using commercially available ELISA kits (R&D, USA) following the manufacturer's instructions. The remainder was snap-frozen for Sircol collagen assay (Biocolor, Accurate Chemical & Scientific Corp.) according to the manufacturer's instructions. Animal experiments were approved by the IACUC of Brown University in accordance with federal guidelines.

### Statistical analysis

All statistical analysis was performed on GraphPad Prism 9.0.0 (GraphPad Software Inc., USA) unless otherwise noted. Graphing was performed on GraphPad Prism 9.0.0 or OriginLab (v10.1.0.178; OriginLab, USA).

## Results and discussion

Dynamic light scattering (DLS) revealed the base uncoated nanoparticles to have a hydrodynamic diameter of 140.9 ± 0.9 nm, with a negatively-charged surface potential (−19.3 ± 0.05 mV) and a low polydispersity index (PDI) of 0.209 ± 0.012 (Table 1). Lyophilization led to an increase in diameter to just under 300 nm (284.5 ± 12.4 nm). This was further increased to 340.5 ± 10.6 nm once the CP coating was applied. Nanoparticles (both polymeric and liposomal) of similar diameters have shown promise in pulmonary drug delivery through the inhalation route, as determined by both deep lung deposition and efficacy (drug effect and localization) studies.^52–57^ For example, Liu and colleagues successfully delivered ciprofloxacin-loaded liposomes of ∼350 nm diameter to mice, while Jinturkar *et al.* developed etoposide and docetaxel-loaded liposomes of 200–350 nm as an inhalable formulation, which were successfully delivered to A549 lung epithelial cells for the treatment of cancer.^53,58^ In the polymeric NP space, work by Sharma *et al.* used lectin-functionalized PLGA NPs of 350–400 nm for delivery *via* the oral/aerosol route, while Puri *et al*. employed inhalable PLGA NPs of approx. 300–315 nm in size to target tuberculosis.^52,57^ Both Jinturkar and Puri have demonstrated that nanoparticles of sizes similar to our formulation (>300 nm) successfully undergo deep lung deposition, bypassing the mucus barrier in particular. Our own previous research on developing inhalable, fluorescent dye-containing PLGA-based NPs has confirmed deep lung deposition and wide-spread fluorescence from the NPs in rat lungs.^39^ Additionally, pectins with a low degree of methylation (DM; <50%) are capable of penetrating mucus- with the pectin used herein having a DM of 6–7%, which will enable our CP-coated NPs to penetrate respiratory mucus easily.^59–61^

**Table 1:** DLS and zeta potential measurements of bare and CP-coated PLGA-NH_2_ nanoparticles before and after lyophilization

Particle synthesis, conjugation and sizing	−CP pre-lyo	−CP post-lyo	+CP post-lyo
Size (nm)	140.9 ± 0.9	284.5 ± 12.4	340.5 ± 10.6
PDI	0.209 ± 0.012	0.350 ± 0.031	0.380 ± 0.042
ZP (mV)	−19.3 ± 0.05	−26.5 ± 0.7	−20.9 ± 0.8

**Fig. 2:**
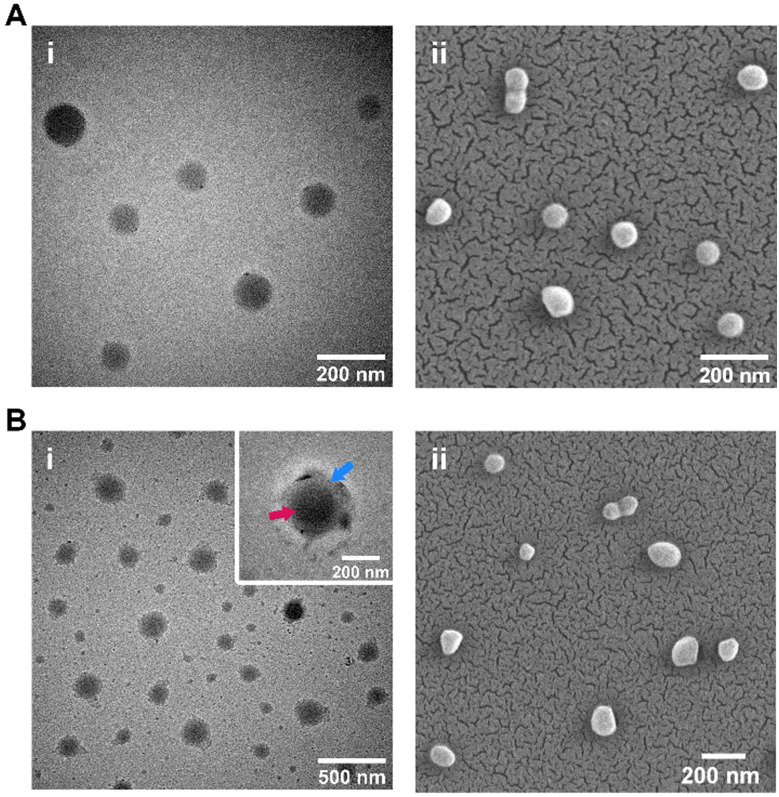
Micrographs of coated and uncoated nanoparticles. (A) Bare/uncoated PLGA-NH_2_ nanoparticles under transmission (i) and scanning (ii) electron microscopy (scale: 200 nm); (B) CP-coated nanoparticles under (i) transmission electron microscopy (with some free CP visible; scale: 500 nm); Inset: high-magnification image of CP coating [blue arrow] on the NP surface [red arrow; scale: 200 nm], and (ii) scanning electron microscopy (scale: 200 nm).

A minor increase in PDI was also observed following conjugation, possibly due to variations in nanoparticle coating thickness. This is borne out by variations observed under TEM imaging (Fig. 2), which showed CP-coated NPs to have a rough, relatively uneven CP ‘halo’ around the central core NP. This is particularly visible on Fig. 2B-i's inset image. Diameters of the nanoparticles as assessed through SEM/TEM imaging reflected the changes in particle size seen in DLS data, with most coated NPs showing an increase in diameter (Fig. 2A-ii*vs.*Fig. 2B-ii). The Zeta potential of the coated and uncoated particles appeared to remain relatively steady in the −20 to −25 mV range (Table 1).

A stability study was conducted to evaluate the particles’ behaviour in Gamble's solution/simulated lung fluid (SLF), with PBS being included for comparison. The former models the interstitial fluid of the deep lung that these particles are expected to interact with once administered *in vivo*, making it a useful medium to assess if the NPs would show any signs of aggregation or other effects that would alter their expected behaviour as nanoscale particles. The results of this study (see the ESI,† Fig. S3) showed the CP-PLGA NPs underwent swelling up to 140% of day 1 size in SLF (compared to approx. 130% in PBS), while ZP stayed relatively constant in SLF (a 5 mV increase was observed in PBS). No major signs of aggregation or agglomeration were observed, and the NPs were stable over the period assayed. Differences between ZP in Table 1 and Fig. S3 (ESI†) are attributable to the different environments in which it was measured in both: ultrapure water in the former, PBS/SLF in the latter. No loss of CP from the NP surface was expected due to the covalent conjugation employed and was not observed to occur in any detectible way as assayed through FT-IR (data not shown). Carbodiimide-mediated conjugation has been an established method of creating strong and highly stable conjugated molecules for a wide variety of *in vitro* and *in vivo* applications (including drug-antibody conjugates, fluorescent probes, or for delicate work such as biosensing).^62–67^ The CP coating is therefore not expected to experience any notable slough-off until well absorbed by cells of the respiratory system.

The conjugation of CP to the pre-made PLGA-NH_2_ NPs was assessed through four methods: TEM, UV-vis spectrometry, FT-IR spectroscopy and TGA. As noted previously, TEM imaging clearly showed a core–shell structure that comprised the coated NPs, indicating successful coating of bare NPs. Quantification of conjugated CP by means of absorbance measurements revealed an average conjugation efficiency (*i.e*., percentage of CP found to have undergone conjugation to PLGA) of 40.3% (some batch-to-batch variation exists; approx. range = 30–50%). Given the 1 : 1 ratio of CP : NPs used during conjugation, this points to a sample composition of approximately 2 : 3 CP : PLGA by weight.

**Fig. 3:**
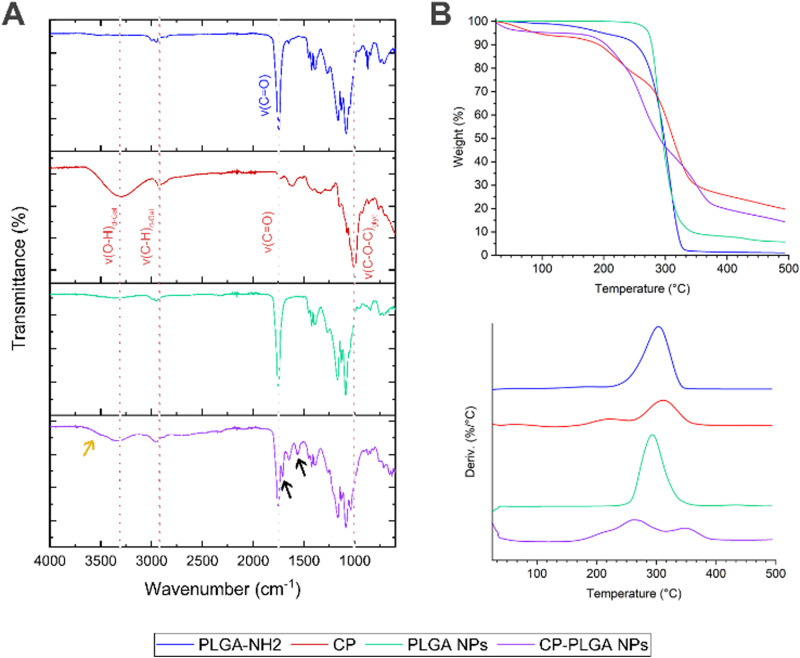
Confirmation of CP conjugation to PLGA NPs by way of (A) FT-IR spectroscopy and (B) TGA (top: thermogram; bottom: first derivatives). FT-IR spectra clearly show the incorporation of CP into the coated PLGA NP structure, with arrows in black highlighting the amide I and II bands that form as a result of the reaction between PLGA's –NH_2_ groups and CP's –COOH groups. Arrow in orange indicate possible amide A band as a shoulder of the *ν*(O–H) peak. TGA shows novel thermal decomposition properties of CP-PLGA nanocomposite, with three-step decomposition profile.

FT-IR spectroscopy (Fig. 3A) of the amine-terminated PLGA showed multiple characteristic transmittance bands expected for the polymer's structure: the band at approx. 3500 cm^−1^ is likely attributable to the stretching of terminal O–H bonds which, being present only in trace quantities (relative to the long polymer chains), produce a broad but very weak signal. A similar weak transmittance signal at approx. 2850 cm^−1^ can be attributed to the stretching of C–H bonds within the polymer, with some possible contribution from N–H stretching of the terminal amine groups of the polymer chains; bending vibrations of the N–H bonds are reflected in the transmittance band at approx. 1650 cm^−1^.^68^ The presence of the ubiquitous C=O bonds within the body of the polymer chain is reflected by the transmittance band at approx. 1750 cm^−1^.^68,69^ This last is conserved in the spectra of all three samples containing PLGA: namely, the polymer itself, the bare NPs and the CP-conjugated NPs. The transmittance spectrum of pure CP shows the presence of numerous –OH groups (found as part of the polysaccharide's galacturonic subunits) by way of a broad, medium strength band at approx. 3300 cm^−1^, and characteristic C–H stretching at 2926 cm^−1^.^70^ The triple band system in the 1738–1517 cm^−1^ region has been previously observed in pectins, being attributed to the C=O stretch of methyl ester groups (1738 cm^−1^) and asymmetric (1612 cm^−1^) and symmetric (1517 cm^−1^) C=O stretching of carboxylate groups.^70,71^ The strong absorbance bands at 1076 and 1009 cm^−1^ have been attributed to glycosidic bonds between the sugar monomers.^72^ The final, CP-coated/conjugated NP sample bears many hallmarks of both the pure PLGA and the CP: the O–H bond stretch from CP is apparent at 3330 cm^−1^, as is the C–H stretch (shifted slightly to 2950 cm^−1^); the C=O contribution from the polymer is also readily apparent at 1750 cm^−1^, as is the presence of glycosidic bonds (albeit with a slight shift). Of particular importance, however, is the appearance of two new transmittance bands: the characteristic amide I & II bands at 1650 cm^−1^ (indicative of an amide C=O stretch) and 1564 cm^−1^ (caused by a mixture of amide N–H and C–N bond stretching) respectively (indicated by black arrows on Fig. 3A).^73^ These above all confirm the establishment of amide bonds on the surface of the NPs, and together with the overlap of spectra observed in the final sample, indicate successful conjugation of CP to the surface of the pre-formed PLGA NPs.

Thermogravimetric analysis (Fig. 3B) revealed an intriguing phenomenon: while neat PLGA and bare PLGA NPs exhibit their characteristic^74^ single-step thermal degradation/mass loss profile (CP showing two, in agreement with the literature^75,76^), the CP-PLGA NPs exhibit a three-step pattern (summarized in Table 2). Two of these three weight losses peak at temperatures significantly different from those exhibited by the formulation's individual components, indicating the formation of a novel, thermally distinct, composite. This phenomenon has been observed in certain cases of copolymer synthesis: Chan and colleagues synthesized pectin–poly(ethylene glycol) methacrylate hydrogels, which exhibited a distinctly two-step thermal degradation pattern of the product despite the components having just one step (ignoring moisture loss).^77^ Moreover, the composite appears to be more thermally stable when compared to its components: manually-calculated extrapolated onset temperatures (*T*_o_) for PLGA NPs and CP were 274.2 and 178.9 °C respectively, while that of CP-PLGA NPs was 184.0 °C, with the final weight-loss maximum being 30–40 °C higher than either constituent, at 348.0 °C. The results of the TGA not only confirms the presence of the CP on the CP-PLGA nanoparticles, but also indicates the successful chemical conjugation of the PLGA and CP by way of the significantly different thermal profile of the end-product. Finally, the TGA also indicates that aside from a small loss of likely loose moisture from the CP component at temperatures approaching 40–50 °C, the CP-PLGA NPs are thermally stable up to about 184 °C.

**Table 2:** Summary of thermal degradation temperatures of CP-PLGA NPs and their neat components as measured by TGA

	PLGA	CP	PLGA NPs	CP-PLGA NPs
Peak 1 (°C)	303.0	213.5	293.0	209.0
Peak 2 (°C)	—	312.0	—	263.0
Peak 3 (°C)	—	—	—	348.0

### Evaluation of cytocompatibility

**Fig. 4:**
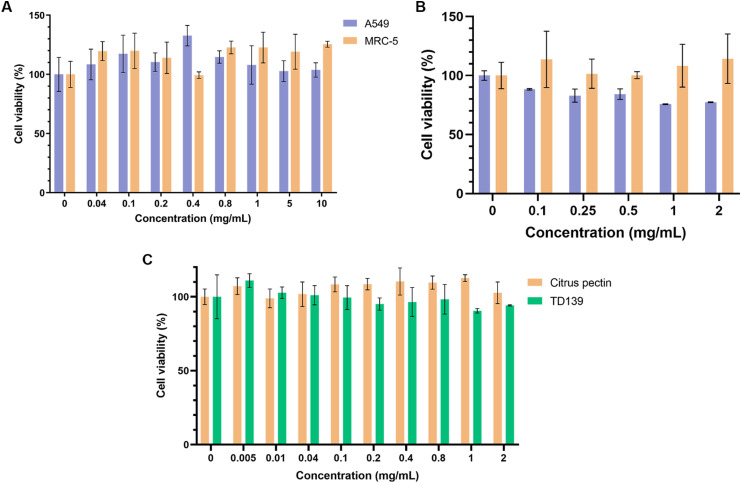
Viability of cells after 24-hour treatment with (A) free citrus pectin (on both A549 and MRC-5 cells), (B) CP-PLGA NPs (on A549 and MRC-5), and (C) citrus pectin or TD139 on MRC-5s (*n* = 4).

Preliminary studies assessing the viability of human lung fibroblasts (MRC-5) and model lung epithelial cells (A549) following 24-hour treatment with up to 10 mg mL^−1^ of CP showed no signs of significant cytotoxicity (Fig. 4A). While there is a large body of evidence pointing to some cytotoxicity of heat-/enzyme-/pH-modified CP against various cancer cell lines, the cytocompatibility of unmodified CP with healthy cells appears to be high.^78–80^ CP-PLGA NPs were also assessed for their cytocompatibility with the same cell lines as above (Fig. 4B). The results confirmed that the cells were viable up to 2 mg mL^−1^ in both types of cells. Although the cancer cell line, A549, exhibited a slight decrease in cell viability at the higher concentrations, >80% cells were viable at these concentrations confirming that the formulation is cytocompatible. This slightly decreased viability in A549 cells is likely the result of two factors: improved cell penetration by nanoscale CP as compared to bulk CP, and the aforementioned anti-cancer effects of citrus-derived pectins. Nevertheless, the most notable result here is that the cell population targeted–lung fibroblasts–appear to show no adverse reaction to either bulk or nanoscale CP. This implies that these cells are left unharmed during the exertion of the therapeutic effect of the NPs.

Fig. 4C demonstrates that CP's cytocompatibility in lung fibroblasts is comparable to that of TD-139, another gal-3 inhibitor previously assessed for PF treatment, exhibiting no major cytotoxicity up to 2 mg mL^−1^.

### Evaluation of *in vitro* uptake and targeting efficacy

**Fig. 5:**
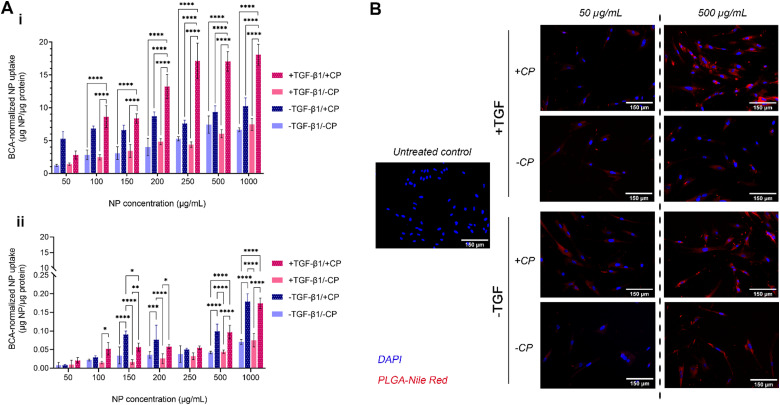
(A) Quantitative assessment of 2-hour uptake of bare- and CP-coated PLGA NPs (containing Nile Red dye) by: (i) MRC-5 fibroblasts and (ii) A549 model epithelial cells at concentrations from 50- to 1000 μg mL^−1^. Nanoparticle uptake has been normalized by means of cell protein content (*n* = 4; **** *P* ≤ 0.0001); (B) representative EVOS microscopy images of NP uptake by MRC-5 cells (scale bars: 150 μm).

The uptake of our formulation by MRC-5 cells (both with and without the CP coating, as well as with and without induced fibrosis in target cells) was measured over a period of 2 hours (Fig. 5A-i). MRC-5 cells with TGFβ treatment (commonly at 5 ng mL^−1^) have been widely studied as an established *in vitro* model of pulmonary fibrosis.^81–83^ The results show a mostly dose-dependent pattern of uptake with two notable exceptions: CP-coated particles administered to both fibrotic and non-fibrotic cells. In the case of the former, uptake at the lowest tested concentration (50 μg mL^−1^) is lower than that of CP-coated particles administered to non-fibrotic cells; uptake then increases in a roughly dose-dependent pattern until 250 μg mL^−1^, when it plateaus, indicating a saturation effect. In the case of the latter, uptake seems to be high even at the lowest tested concentration and rises by less than 2-fold despite a 20-fold increase in administered concentration, exhibiting a plateaued uptake at concentrations of about 100–200 μg mL^−1^. Taken together, these observations on CP-coated NPs seem to indicate that CP coating enhances general uptake by fibroblasts. While the uptake of CP-PLGA NPs by non-fibrotic cells at lower concentrations may indicate the possibility of off-target accumulation (but unlikely to be toxic, given the cytocompatibility data), this is only apparent at sub-200 μg mL^−1^ concentrations: the uptake of CP-PLGA NPs by fibrotic MRC-5s becomes statistically significant (*p* < 0.0001) against all other tested experimental groups above this concentration. These trends are also observed in qualitative data presented in Fig. 5B, reinforcing the patten of uptake seen in the quantitative assays.

The dose-dependent uptake observed in lung fibroblasts is to be expected given the affinity of pectins to gal-3. The interaction between the β-galactoside-binding carbohydrate recognition domain (∼14 kDa region) of gal-3 and the β-galactoside-rich CP is generally known to possess a dissociation constant (*K*_D_) in the range of 50–100 μM.^26,84–87^ With the ubiquitous expression of gal-3 on the surface of fibroblasts (among other pulmonary cell populations), and the upregulation of this expression during fibrosis, the increased binding of CP (aided by its nanoscale presentation in this case) during TGFβ-induced fibrosis and with increasing CP-PLGA NP dose fits in with the behaviour predicted by extant literature.^17–21^ Evaluation of NP uptake by seeding density-matched model pulmonary epithelial cells (A549s; Fig. 5A-ii) showed uptake by these cells occurred at an order of magnitude lower than fibroblasts, with epithelial cells exposed to pro-fibrotic TGFβ taking up more of the particles than non-exposed controls. In effect, we demonstrate here that our CP-coated PLGA NP platform is able to target fibrotic cells with limited impact on off-target cell subpopulations.

### *In vitro* anti-fibrotic efficacy

**Fig. 6:**
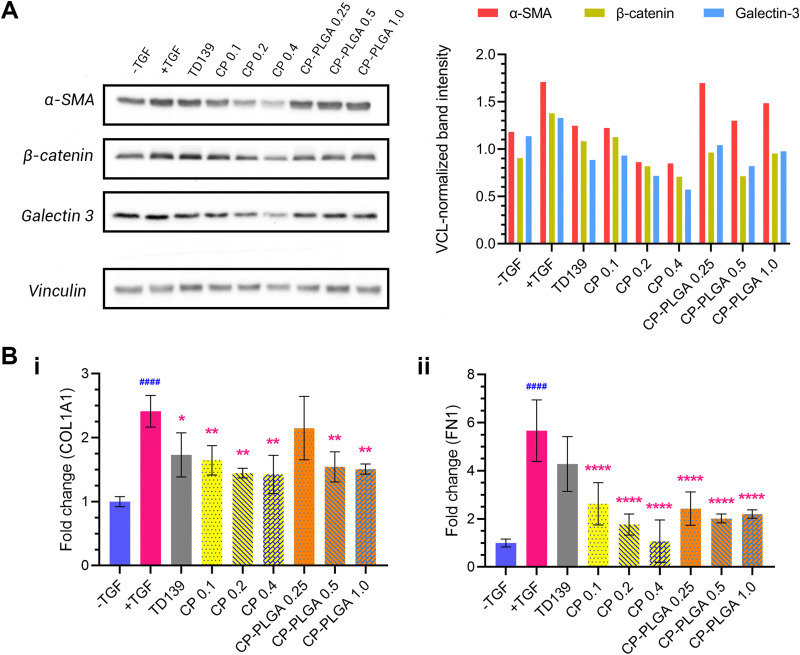
(A) Western blot (and quantification thereof) of 3 key markers of fibrosis in MRC-5 cells, 24 h incubation with single dose of treatments listed. All groups (except the -TGF control) were concurrently treated with 5 ng mL^−1^ TGFβ. Free CP was run at 0.1–0.4 mg mL^−1^, while CP-PLGA NPs were run at 0.25–1.0 mg mL^−1^. A general trend of reduction (relative to +TGF-only group) were seen for free CP and CP-PLGA NPs at most concentrations tested, with the exception of 0.25 mg mL^−1^ CP-PLGA. (B) Results of RT-PCR quantification of (i) COL1A1 and (ii) FN1 mRNA in MRC-5 cells subjected to treatment as in (A). Statistically significant downregulation of COL1A1 compared to the +TGF-only group was observed in all treatment groups except 0.25 mg mL^−1^ CP-PLGA, while TD139 was the sole exception in the case of FN1 (indicated by red asterisks (*); blue hashes (^#^) indicate significance between healthy and fibrotic controls) (* *P* ≤ 0.05, ** *P* ≤ 0.01, ****/^####^*P* ≤ 0.0001; *n* = 3).

An evaluation of the effect of CP-PLGA NPs on fibrotic human lung fibroblasts was carried out *via* western blotting, and the results are presented in Fig. 6A. Treatment with TGFβ caused an elevation of all markers tested (relative to untreated, healthy controls): concurrent administration of therapeutic groups (TD-139, CP and CP-PLGA) alongside TGF treatment for 24 hours caused a clear, mostly dose-dependent decline in the expression of all markers. Raw PLGA NPs were not evaluated at this stage, as they have no documented propensity for the reduction of any known fibrotic markers.^88,89^ The choice of free CP concentrations here was based on the approximately 40% CP (w/w) composition of the final CP-PLGA formulation: 40% of the concentrations of CP-PLGA NPs trialled were thus employed for the free CP groups.

TD-139, included as a benchmark as mentioned previously, induced an appreciable decrease in marker expression relative to the TGFβ-only group. Free CP showed a strong anti-fibrotic effect at concentrations above 0.1 mg mL^−1^ (which itself had an effect comparable to that of TD-139), particularly against α-SMA. This trend was reflected in the CP-PLGA NPs as well, albeit to a slightly lesser extent. Nevertheless, CP-PLGA at 0.5 mg mL^−1^ appeared to induce a notable decrease in expression of all markers tested after just 24 hours’ incubation, coming close to the reduction observed in the TD-139 group. The anti-fibrotic effects of CP and CP-loaded PLGA NPs were robust, yet not always dose-dependent, which aligns with previous findings by Abu-Elsaad *et al*. and Yin *et al*.^90,91^ This absence of dose-dependency may be attributed to multiple factors. First, the large molecular weight of CP could limit its uptake at higher concentrations, leading to inconsistent responses. Additionally, CP may operate through a non-linear mechanism of action, where its therapeutic effects do not increase with higher doses. Future studies will include a detailed evaluation of varying concentrations of CP and CP-coated NPs both *in vitro* and *in vivo* to further explore these observations. As there was no dedicated anti-fibrotic drug encapsulated within our formulation, the observed therapeutic effect can be attributed entirely to the CP coating. This is also true for the patterns of expression of COL1A1 and fibronectin (FN1) mRNA observed in RT-PCR studies (Fig. 6B), where free CP and CP-PLGA NPs caused marked, statistically significant attenuations in the upregulation of both. The more marked reductions here were to be expected, given the more transient (and thus more readily affected) nature of mRNA as compared to the slower downregulatory effect on the levels of pre-formed proteins. In the case of COL1A1, all three concentrations of free CP appeared to cause a roughly similar effect on expression, alongside TD139, while a significant effect was observed in only the two higher concentrations of CP-PLGA tested, albeit of a lower magnitude. Conversely, TGF β-mediated upregulation of FN1 was not attenuated by TD139, while all CP-containing groups did, again with CP-PLGA having a less noticeable effect than free CP. While levels of COL3A1 mRNA were assessed in a similar way, no clear trend was noticeable unless at very high concentrations of CP-containing groups (see the ESI,† Fig. S4). Citrus-derived pectins have previously been shown to be capable of downregulating the expression of fibronectin and collagens 1 and 3 caused by fibrosis, and the results presented herein appear to, broadly, follow this established pattern.^28,30,90^

While free CP demonstrated comparable or even superior anti-fibrotic effects than CP-PLGA NPs *in vitro*, where cells were directly exposed to the treatment groups, we anticipated greater anti-fibrotic effects from CP-coated NPs following inhalation *in vivo*. This expectation was based on the likelihood that inhaled free CP would not achieve sufficient levels in the deep lung tissue. As shown by us previously, inhaled NPs are expected to deposit more extensively in the deep lung, achieving more significant therapeutic outcomes.^39,92^ When combined with encapsulated NIN or PFD, there is a high likelihood, therefore, that this platform can serve to deliver a two-pronged anti-fibrotic effect: a more rapid initial one from the action of the surface-conjugated CP, followed by a sustained, longer-term effect as a result of encapsulant release from the PLGA matrix. Coupled with the CP's ability to help the NPs target fibrotic tissue *via* the CP-gal-3 interaction (as demonstrated in Fig. 5), this platform may serve to improve anti-fibrotic therapeutic efficacy by both avoiding off-target effects common to these drugs, and by the combined effect of CP and the NIN/PFD. This is now a promising avenue of research we envision following in the future.

### *In vivo* anti-fibrotic activity

**Fig. 7:**
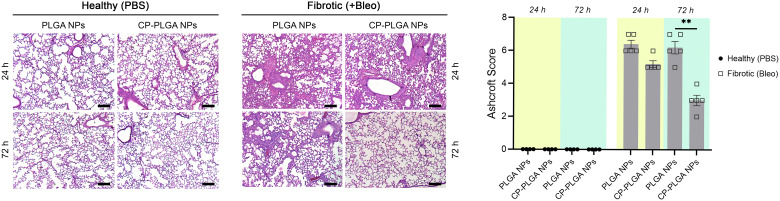
H&E staining of lung tissue sections (all scale bars: 200 μm) of mice administered either PBS (vehicle control) or bleomycin (Bleo; induced fibrosis), followed by administration of either bare or CP-coated PLGA NPs. Note the clear fibrogenesis in the mice administered bleomycin, dissipating within 72 hours of CP-PLGA administration.

**Fig. 8:**
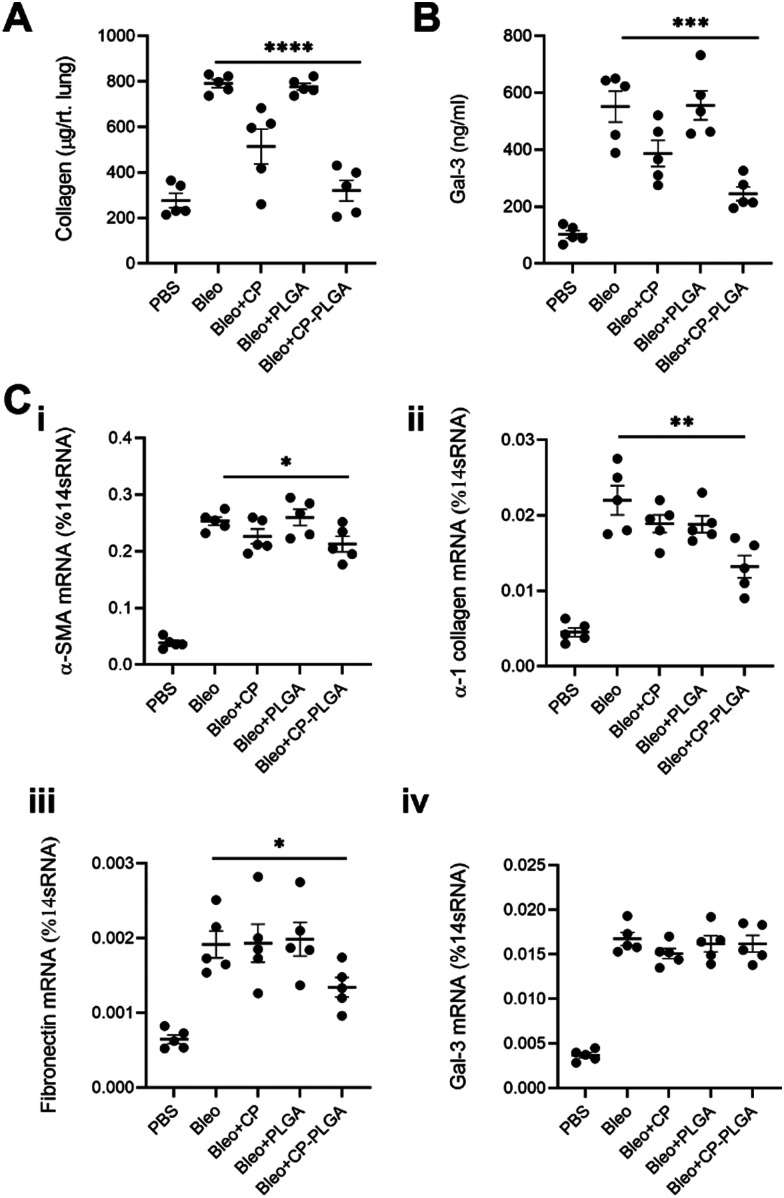
*In vivo* efficacy of CP-PLGA NPs as measured by (A) sircol collagen assay – whole lung, (B) galectin-3 ELISA – whole lung, and (C) RT-PCR for: (i) ACTA2, (ii) COL1A1, (iii) FN1 and (iv) LGALS3 (* *P* ≤ 0.05, ** *P* ≤ 0.01, *** *P* ≤ 0.001, **** *P* ≤ 0.0001; *n* = 5).

Lung tissue from healthy or (bleomycin-administered) fibrotic mice were excised and subjected to H&E staining 24- and 72-hours after administration of either bare- or CP-coated PLGA NPs (Fig. 7). Bleomycin induced significant fibrosis within the lungs, with visible thickening of alveolar septa, collapse of alveolar spaces, and significant infiltration of fibroblasts and inflammatory cells. No changes were observed in the lungs of healthy controls (whether administered with PLGA or CP-PLGA NPs) at either timepoints, further reinforcing the safety of CP's use *in vivo* and the lack of any deleterious effects on healthy cells or tissue. The evaluation of the final formulation's efficacy in diseased mice, however, showed a clear and marked reduction in fibrosis at the 72 h timepoint. PLGA NPs, conversely, had no impact on the degree of fibrosis. These changes were reflected in the Ashcroft scores presented in Fig. 7, which showed a non-significant decrease at 24 h (bare *vs.* coated NPs; a trend matching *in vitro* data) and a significant decrease at 72 h. This reduction of fibrotic hallmarks was reflected in more in-depth studies carried out over a longer time period (7 days post-treatment), performed *via* direct measurement of pro-fibrotic markers in whole-lung samples, presented in Fig. 8. Free CP's effects were not evaluated as part of Fig. 7 as such data has been previously-published and shown to be consistent with trends observed in Fig. 6 and 8.^28,93^ Xu and colleagues, for instance, used CP to treat isoproterenol-induced heart failure in male Wister rats.^28^ H&E staining carried out by them 15 days following pectin administration showed marked reduction of fibrotic structural changes in myocardial tissue. This extended to significant reduction in the percentage area of collagen deposits seen in cardiac tissue. Similarly, Mahmoud *et al.* administered pectin to male Wistar rats with type 2 diabetes, over a course of 4 weeks.^93^ Their H&E staining images also showed significant inhibition of renal fibrosis secondary to diabetic nephropathy, and collagen and fibrous tissue observed in diseased controls were completely missing from kidneys of pectin-administered animals.

Collected 7 days post-administration of the various treatments, these whole-lung samples showed statistically significant reductions in collagen (both in terms of protein content and mRNA levels), α-SMA, and fibronectin (Fig. 8A and C-i to iii). These levels followed a fairly predictable and expected pattern: increased marker expression in bleo-administered animals, with significant reductions in both the free CP and CP-PLGA groups, and none in the PLGA-only group. This is in keeping with the effect of gal-3 inhibitors on levels of collagen observed by other groups: Xu and colleagues in particular noted strong reductions in collagen expression and deposition in the context of myocardial fibrosis, upon treatment with CP.^28,30^ The magnitude of reduction induced by the nanoformulation varied, however: while absolute levels of collagen were reduced markedly relative to the diseased controls, the rest did not experience as drastic a decrease in each case. Gal-3 was of particular interest, not least due to its interaction with CP: levels of mRNA for LGALS3 did not appear to change significantly once bleomycin administration was complete (Fig. 8C-iv). This may point to CP's possible interference in the post-translational fate of gal-3, although longer-term studies would be required to confirm if the silencing of gal-3 expression happens at more extended timescales than those assayed herein. This prompted an investigation into levels of gal-3 in these whole-lung samples (Fig. 8B), which showed an approximate halving in the CP-PLGA treatment group, relative to bleo-only controls. While our H&E staining images at 72 hours demonstrates the initial anti-fibrotic effects of the CP-PLGA NPs, the data presented in Fig. 8 at 7 days confirms continued suppression of pro-fibrotic markers in the lungs, indicating that the therapeutic effects of the NPs are sustained over time. These results support the potential use of CP-PLGA NPs for long-term therapeutic benefit in pulmonary fibrosis.

The *in vivo* trends observed do not correlate exactly with those seen in an *in vitro* context, where free CP had a greater effect in reducing fibrotic markers. A fact of note is that free pectins and other drugs delivered by inhalation are known to have a half-life in the order of several hours, compared to the much longer lung residence times of PLGA-based nanomaterials (order of days) observed in previous work by our group and others.^39,41^ As such, a more pronounced antifibrotic effect is expected to be observed *in vitro* as compared to *in vivo*, with the former lacking any means of clearance and cells being exposed to free CP for much longer than they would be *in vivo*. By contrast, we expected CP-PLGA NPs to exert a similar or greater effect compared to free CP *in vivo* due to the aforementioned longer half-life/clearance time, and this was indeed reflected in the data shown in Fig. 8.

Overall, our *in vivo* data reinforces our *in vitro* data and demonstrates the high degree of anti-fibrotic activity of the CP-PLGA platform. The inclusion of a dedicated anti-fibrotic therapeutic within the PLGA core is expected to work in tandem with this effect, enhancing therapy through both broadly cell-specific targeting and a cumulative anti-fibrotic effect.

## Conclusions and future work

Pulmonary fibrosis is a condition for which there is still a wide expanse of open avenues for research in terms of both developing novel therapeutics and in improving currently available ones. Attempting to address the latter, we report here a novel, targeted and inherently anti-fibrotic nanoparticle platform by conjugation of pectin from citrus peel (CP) to a PLGA-based core, synthesizing a hybrid copolymer nanoparticle (CP-PLGA NPs) in the process. While we have shown that this formulation holds great promise in acting as a platform for delivering an anti-fibrotic payload, some limitations do exist. The large molecular weight of CP may be responsible for the 300+ nm diameter and batch variability observed in the final CP-PLGA NP formulation. To further optimize these, modified CP may be considered as an alternative. Also, the work described herein is a preliminary study; we have not attempted to explore therapeutic response over extended time scales beyond a maximum of 7 days *in vivo* (or examine how the effect of these NPs impact lung function) or encapsulated anti-fibrotic agents. We hope to investigate the effects of these formulations over longer time periods as part of future work, whereintransgenic mouse models and FITC-induced murine models of pulmonary fibrosis may be employed.^94,95^ In addition, studies to examine gender-based variability in anti-fibrotic therapeutic response may be carried out, as such differences have been observed with existing anti-fibrotics in literature.^96–99^

Nevertheless, the CP-PLGA NPs were shown to be capable of being selectively taken up by fibrotic MRC-5 cells, were well-tolerated by them, and induced a marked reduction in markers such as α-smooth muscle actin, β-catenin, and gal-3 *in vitro*, as well as statistically significant reductions in the expression of COL1A1, gal-3 and fibronectin *in vivo* in a murine bleomycin-induced model of pulmonary fibrosis. In this work, we present our CP-PLGA NPs as a proof-of-concept for a dual action (targeting + therapeutic) delivery vehicle and lay the groundwork for exploring the potential of CP-PLGA NPs with encapsulated anti-fibrotics in the future, in the hope of enhancing the range of available options to treat pulmonary fibrosis.

## Author contributions

KP: investigation, methodology, formal analysis, writing – original draft, writing – review & editing; MG: investigation, methodology, formal analysis, writing – original draft; PS: investigation, methodology, formal analysis, CN: investigation, formal analysis; SN: investigation; YZ: funding acquisition, resources, supervision, writing – review & editing; JUM: conceptualization, methodology, funding acquisition, resources, supervision, writing – review & editing.

## Data availability

Most of the data supporting this article are available within the article and as part of the ESI.† Due to confidentiality, any data not presented here can be made available subject to a non-disclosure agreement.

## Conflicts of interest

There are no conflicts to declare.

## Supplementary Material

TB-013-D4TB01682C-s001
