# Supplementary material for: Citrus pectin-coated inhalable PLGA nanoparticles for treatment of pulmonary fibrosis
Source: J Mater Chem B. 2025 Jan 27;13(10):3325–39. doi: 10.1039/d4tb01682c (PMC11804936; doi:10.1039/d4tb01682c)
Supplement: TB-013-D4TB01682C-s001 [file TB-013-D4TB01682C-s001.pdf]

Supplementary Information (SI) for Journal of Materials Chemistry B.
This journal is © The Royal Society of Chemistry 2025

Supplementary Information for:

Citrus Pectin-Coated Inhalable PLGA Nanoparticles for Treatment of Pulmonary Fibrosis

Kalindu Perera, Moez Ghumman, Parand Sorkhdini, Carmelissa Norbrun, Seraphina Negash, Yang Zhou*, Jyothi U. Menon*

(*Corresponding authors: jmenon@uri.edu, yang_zhou@brown.edu)

Table S1 Primer sequences used for RT-PCR.

Experiments	Gene	Primers
In vitro (human)	COL1A1	F: GAGGGCCAAGACGAAGACATC<br>R: CAGATCACGTCATCGCACAAC
	COL3A1	F: CCACTTGGGATTGCTGGGAT<br>R: GGACCACGTTCTCCACTGAG
	FN1	F: CGGTGGCTGTCAGTCAAAG<br>R: AAACCTCGGCTTCCTCCATAA
In vivo (mouse)	ACTA2	F: GTCCCAGACATCAGGGAGTAA<br>R: TCGGATACTTCAGCGTCAGGA
	COL1A1	F: GCTCCTCTTAGGGGCCACT<br>R: CCACGTCTCACCATTGGGG
	FN1	F: ATGTGGACCCCTCCTGATAGT<br>R: GCCCAGTGATTTCAGCAAAGG
	LGALS3	F: AGACAGCTTTTCGCTTAACGA<br>R: GGGTAGGCACTAGGAGGAGC

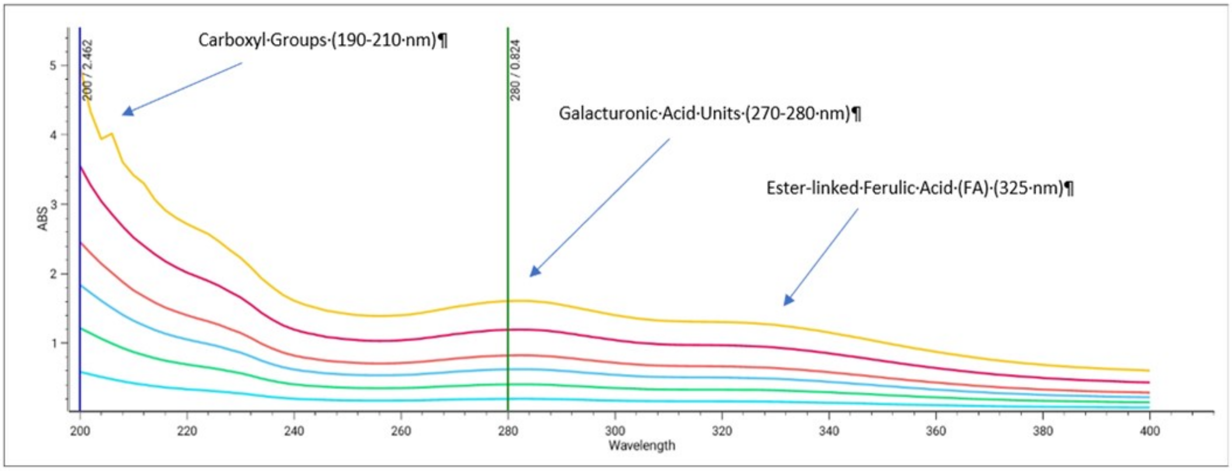

Fig. S1 Graph of UV-Vis spectrophotometric spectral wavelength scan of citrus pectin at 0.25- (light blue), 0.5- (green), 0.75- (blue), 1 (orange), 1.5- (purple), and 2 mg/mL (yellow) concentrations (ascending spectra) from λ= 200-400 nm.

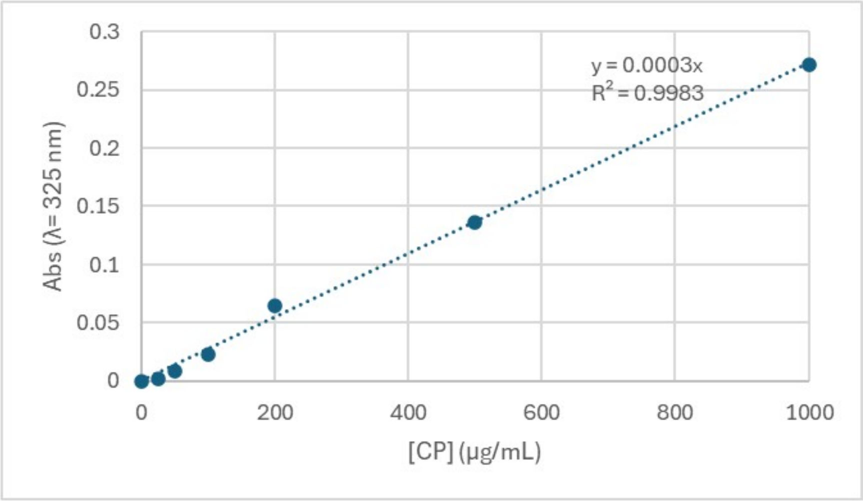

Fig. S2 Calibration plot used for quantification of CP in CP-PLGA NPs, using wavelength identified from S1 above.

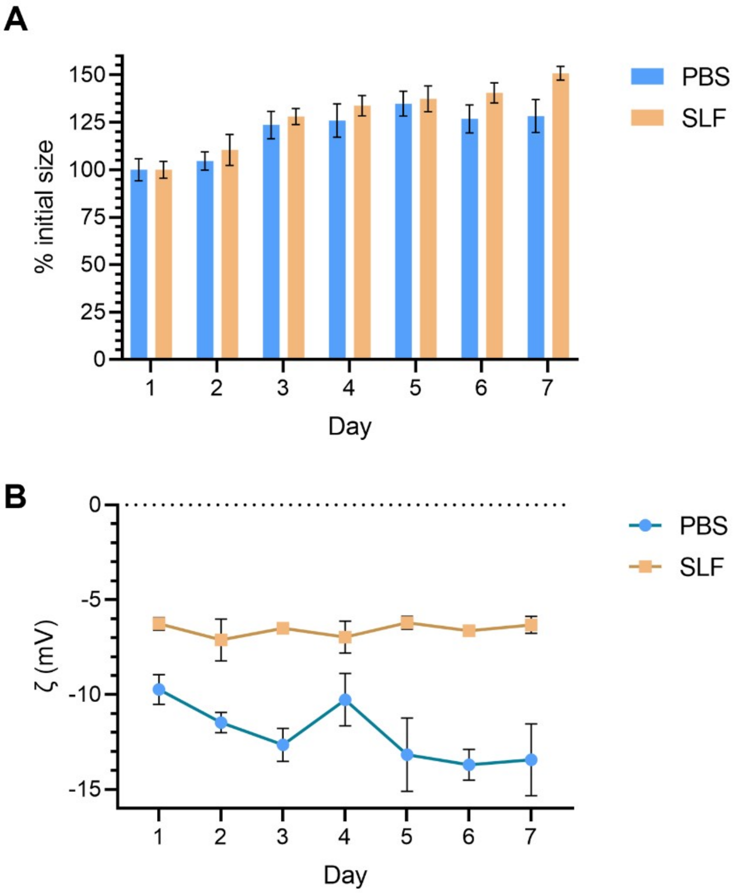

Fig. S3 Results of nanoparticle stability assessment in phosphate buffered saline (PBS; pH 7.4) and simulated lung fluid (SLF), with respect to **A)** size and **B)** zeta potential (ζ). Some swelling of the CP-PLGA NPs was observed over 7 days in both media, while zeta potential tended to remain stable in SLF or get stronger in PBS over the same period.

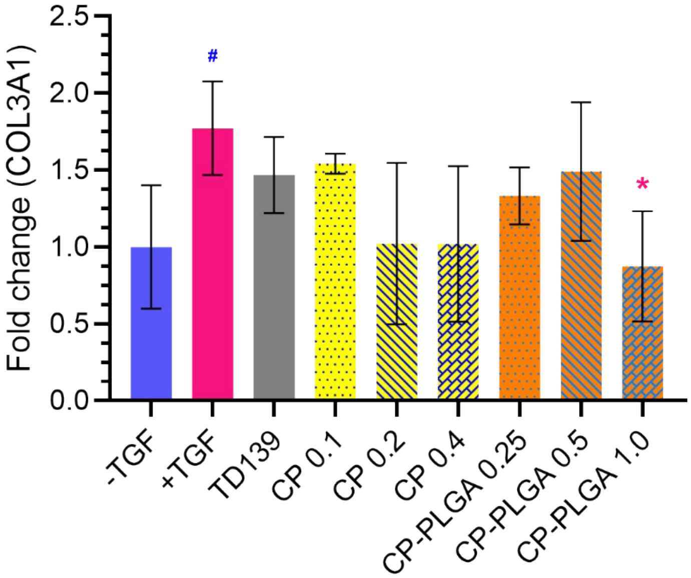

Fig. S4 Results of RT-PCR quantification of COL3A1 in MRC-5 cells subjected to treatment as in Fig. 6 in main text. Statistically significant downregulation of COL3A1 compared to the +TGF-only group was observed at only 1.0 mg/mL CP-PLGA (indicated by red asterisk). (*/# $P \leq 0.05$; n=3).
